# Health Food Company

**Published:** 1875-12

**Authors:** 


### THE HEALTH FOOD COMPANY.

The first article introduced by this company at 137 East Eighth St., New York, was the GRANULATED WHEAT BISCUIT, the sales of which have been very large. They are undoubtedly the best food of the cracker or biscuit kind ever made. They are from sound, ripe, plump wheat, well-cleansed, kiln dried, reduced to a powder by pounding, unsifted, mixed with pure water and baked in soap-stone ovens. No salt, no grease, no yeast, no baking-powders, are used; nothing but wheat and water. The result is a thin, crisp, splendid whole-wheat bread, full of strong nourishment, admirable for the digestive organs, and just the thing for brain-workers, who are the most heavily taxed people in the world.

Another great specialty of the Company, is the COLD-AIR FLOUR. This is the best quality of wheat, reduced, by a new process, to a fine powder, and unbolted. Before this process was invented, no fine flour could be bought containing all the nutriment of the grain. The best part—the nitrogen, the phosphate, and the gluten, adhered to the outer shell or bran, and was sifted out, because no mill-stones could reduce it to a fine powder. It *flattened* under the heavy stones, and the thin scales remained in great quantity in the fine bolting cloth. But this process, by which antagonistic currents of cold, compressed air sieze the wheat and tear it to minute atoms in an instant, has made the infinite division of the entire wheat wholly practicable. In the process of pulverising, there is no heating of the mass, and herein rests much of the superiority of the flavor of the flour. The excellence of bread and cake made from this cold-air flour proves conclusively that the common millstone method of reduction is ruinous to the flavor of the cereals. This cold-air flour is destined to be universally used by and by, to the great advantage of the human race. We have nearly ruined our digestive organs by the employment of starch as food—for white flour is chiefly starch; good for stiffening linen, but wretehed to depend upon as food—and this fine, choice, whole-wheat flour comes in at the opportune moment, to save the race from greater misery.

Another article sold by this Company, is PEARLED WHEAT. This is the kind of wheat used in making the cold-air flour. It has the outer hull removed. It is used as rice is used. But it is sweeter and more appetising than rice, by far. Rice is insipid, and only becomes palatable when sweetened and seasoned. As food, to supply the waste of the body, and keep the machine in active motion, it is worth a hundred times more than rice.

It should be well cooked, in a double boiler, with plenty of water. People who think cracked-wheat and crushed-wheat coarse and unpleasant, delight in this Pearled Wheat food, which is free from all harshness of texture, and lacks the slightly acrid taste of the unhulled grain. The Health Food Co. have the best double boilers for cooking this and similar foods to be found in the market.

Still, another food introduced by the Health Food Co. is POMARIUS. This is merely the juice of sound, ripe, tart eating-apples, filtered to the clearness of wine and then reduced 90 per cent. by evaporation in porcelain pans. The product is a heavy jelly, amply sweet, though containing no sugar other than the sugar of the apple, delicately tart and thoroughly delicious. Of course it is wholesome, for pure apple-juice could not be otherwise. It aids digestion, and is therefore grateful to delicate stomachs. If given to children, with the Granulated Wheat Biscuit, the bread made from the COLD-AIR FLOUR, and the Pearled Wheat, they will be strong and healthy, with good bones, good teeth, good complexions, and, above all, good *Brains*.

### PRICES.

GRANULATED WHEAT BISCUIT.—6 lb. boxes, $100; 10 lb boxes, $1.60; barrels holding from 70 to 85 lbs. 15c per lb.

COLD-AIR FLOUR.—$10.50 per barrel; in bags of any size, 6 cents per lb.

PEARLED WHEAT.—In packages of two sizes; 25 cents and 60 cents.

POMARIUS.—In glass, 50 cents and $1.75; the large jar containing precisely 5 lbs. of the jelly.

DOUBLE BOILERS.—Four sizes, in heavy tin, and also in porcelain-lined iron; from $1 to $2.40. The boiler of the largest size holds two quarts.

All the goods of the Health Food Co. are sent per express C. O. D., or by freight, on receipt of price. Cart age is charged on large pakages.

A HINT OR TWO.—In boiling the *Pearled Wheat*, use four times as much water as wheat. It is important that the wheat be thoroughly cooked, as wheat is not good food, if only half cooked. To secure thorough cooking, the heat must be carried to about 212°. This cannot be done with fresh water in a double boiler and no other should be used; because in any other you are very likely to burn the wheat, and if you burn it you utterly ruin it for digestion, therefore you must put a spoonful of salt in the outer boiler. To carry salt water up to the boiling point requires more heat; and thus you secure ample cooking for your excellent food.

### HOW THE NEW FLOUR IS USED.

The new flour differs from that in common use, and requires different treatment. It is full of nutriment, and therefore you cannot eat as much of it. It is over four times as strong in food-value as the best white flour; that is to say, it would be as cheap at $40 a barrel as white flour would at $10. Being rich in gluten, it demands a great deal of water in mixing, as it swells greatly. It should never be kneaded but only stirred.

A Lady sends us this recipe, with some delicious Gems made according to it: Two eggs well beaten; two coffee-cups sweet milk; one teaspoon salt. All the ingredients should be cold. Stir quickly, and bake in iron Gem-pans, in a hot oven, about twenty minutes, or until they are a light brown. Heat the pans while mixing the Gems. Hot pans and a hot oven are absolutely necessary in making this recipe a success.

